# A novel adenoviral vector carrying an all-in-one Tet-On system with an autoregulatory loop for tight, inducible transgene expression

**DOI:** 10.1186/s12896-015-0121-4

**Published:** 2015-02-13

**Authors:** Hao Chen, Dongyang Wang, Ruiting Xia, Qinwen Mao, Haibin Xia

**Affiliations:** Laboratory of Gene Therapy, Department of Biochemistry, College of Life Sciences, Shaanxi Normal University, Xi’an, 710062 Shaanxi PR China; College of Liberal Arts and Sciences, University of Illinois at Urbana-Champaign, Urbana, IL 61801 USA; Department of Pathology, Northwestern University Feinberg School of Medicine, Chicago, IL 60611 USA

**Keywords:** Adenoviral vector, Tet-On, Transgene, Inducible vector, Gene therapy

## Abstract

**Background:**

One of the most commonly used vectors for gene therapy is the adenoviral vector; its ability to tightly regulate transgene expression is critical for optimizing therapeutic outcomes. The tetracycline-regulated system (especially the Tet-On system) for gene expression is one of the most valuable tools for controlling gene expression. The major problem of an adenoviral vector carrying a Tet-On system is suboptimal regulation of transgene expression.

**Results:**

We constructed a single adenoviral vector carrying in its E1 region a novel “all-in-one” Tet-On system with an autoregulatory loop. This system had improved Dox-inducible gene expression in terms of low basal expression, high induced expression and high responsiveness to Dox. To our knowledge, this is the first reported adenovirus-based, all-in-one Tet-On system with an autoregulatory loop inserted into a single region of adenoviral genome. This system was further tested by inducible expression of soluble tumor necrosis factor-related apoptosis-inducing ligand (sTRAIL). The adenovirus that expressed soluble TRAIL under the control of this novel Tet-On system showed tumor-derived cells inhibitory activity in SW480 cells only under induced conditions.

**Conclusions:**

Our novel, single adenoviral vector carrying in its E1 region an all-in-one Tet-On system with an autoregulatory loop displayed tight regulation of transgene expression *in vitro*. This system has great potential for a variety of applications, including gene therapy and the study of gene function.

## Background

The adenovirus (Ad) vector is one of the most commonly used vectors for gene therapy. The tightly regulated expression of transgenes by adenoviruses is very important for optimizing therapeutic outcomes. Several regulated expression systems have been developed in recent years. The most widely used system in gene therapy investigations is the Tet-inducible system [1-6] with its two variants, Tet-Off [7] and Tet-On [8]. Briefly, in the Tet-Off system, by using the tetracycline transactivator (tTA) protein, transcription is turned off in the presence of tetracycline. In the Tet-On system, by using the reverse Tet transactivator (rtTA) fusion protein, transcription is turned on in the presence of tetracycline. In general, the Tet-On system is preferred over Tet-Off for gene therapeutic applications because its responsiveness is faster and because there is no need for continuous pharmacological treatment after gene expression termination. One of the major problems of the original Tet-On system, the leakiness in the absence of the inducer, was solved by taking advantage of several modified, so-called “second generation” rtTAs (rtTA2S-S2, rtTA2S-M2) [9-11], and co-expression of a Tet-controlled transcriptional silencer (tTS or TetR-KRAB) [12-14].

The being said, if a single Ad vector could carry a Tet-On regulation system, it would be more practical and would facilitate the widespread application of that Ad vector. Some studies used an IRES-mediated bicistronic unit for rtTA and tTS expression in the Ad vector context; however, no consistent results were obtained in terms of the regulational properties of this system [15,16]. Mizuguchi et al. produced an Ad-mediated Tet-On system that contained three heterologous gene-expression cassettes, the gene of interest, rtTA2S-S2, and tTS, in the E1 deletion region, the E3 deletion region, and the region between E4 and 3′ITR, respectively, and displayed improved inducible gene regulation. However, the space for therapeutic genes was compromised in their system [15].

In this study, by utilizing a tetracycline-controlled transcriptional silencer (tTS or TetR-KRAB) and the latest version of rtTA (rtTA2S-M2), we constructed a single adenoviral vector carrying an all-in-one Tet-On system with an autoregulatory loop in the E1 region to improve the regulational properties of the Ad-mediated Tet-On system. This system was further tested by inducible expression of soluble tumor necrosis factor-related apoptosis-inducing ligand (sTRAIL) in SW480 cells and its tumor-derived cells inhibitory activity under induced conditions.

## Methods

### Cell culture

HEK 293, a human embryonic kidney cell line (ATCC, CRL-1573), SW480, a human colon carcinoma cell line (ATCC, CCL-228), and U87 MG, a human glioblastoma cell line (ATCC, HTB-14), were cultured in Dulbecco’s Modified Eagle’s Medium (DMEM) (Gibco, Grand Island, NY) containing 10% (v/v) newborn calf serum (NCS) (Gibco, Grand Island, NY) at 37°C in a 5% CO_2_ atmosphere. CHO-K1, a Chinese hamster ovary cell line (ATCC, CCL-61), was maintained in RPMI 1640 medium (Gibco, Grand Island, NY), supplemented with 10% (v/v) NCS at 37°C in a 5% CO_2_ atmosphere.

### The construction of the Bi-Tet-On system

The plasmid pTRE2pur carrying the TRE-PminCMV element and the plasmid pTet-On containing the rtTA element were purchased from Clontech Laboratories (Mountain View, CA). The vector pLVCT-tTR-KRAB containing the TetR-KRAB element and the vector pLVCT-rtTR-KRAB-2SM2 carrying the rTetR-M2-KRAB were purchased from Addgene (Cambridge, MA). The plasmid pTight-PminCMV, p*Kpn*I-CMV-sv40pA, the pAd5-E1 shuttle and the adenoviral backbone plasmid carrying an eGFP expression cassette in the E3 region were all kept in our lab.

The TetR-KRAB cDNA was obtained by PCR from the pLVCT-tTR-KRAB using a pair of primers, P1: A*ATCGAT*ATGGCTAGATTAGATAAA and P2: T*TCTAGA*TTAAACTGATGATTTGAT. The italic primer sequences indicate *Cla*I and *Xba*I restriction sites at both ends.

The rtTA cDNA was amplified by PCR from the plasmid pTet-On using the primers P3: T*GAATTC*ATGTCTAGATTAGATAAA and P4: *ACTAGT*CTACCCACCGTACTCGTC. The italic primer sequences indicate *EcoR*I and *Spe*I restriction sites at both ends. The rTetR-M2 was obtained by PCR from the plasmid pLVCT-rtTR-KRAB-2SM2 using the following primers: P5: T*GAATTC*ATGGCTAGACTGGACAAGAG and P6: C*AGATCT*CGACCCACTTTCACATTTAA. The primers included *EcoR*I and *Bgl*II restriction sites at both ends. Lastly, the 3 × VP16 region was obtained by PCR using P7: *GGATCC*GGCTCGCCGGCCGACGCCCTTGACGATTTTGACTTAGACATGCTCCCAGCCGATGCCCTTGACGACTTTGACCT and P8: *ACTAGT*TTACCCGGGGAGCATGTCAAGGTCAAAATCGTCAAGAGCGTCAGCAGGCAGCATATCAAGGTCAAAGTCGTCAA. The italic primer sequences indicate *BamH*I and *Spe*I restriction sites, respectively, at both ends. Subsequently, the rtTA-M2 fragment was ligated with 3 × VP16 fragment, and the resultant plasmid was named p-rtTA2S-M2.

The rtTA cDNA was cloned into p*Kpn*I-CMV-sv40pA by *EcoRI* and *SpeI* sites, the obtained plasmid was called p*Kpn*I-CMV-rtTA-sv40pA. rtTA2S-M2 cDNA was inserted into the p*Kpn*I-CMV-sv40pA at *EcoR*I and *Spe*I sites, the resultant plasmid was named p*Kpn*I-CMV-rtTA2S-M2-sv40pA. TetR-KRAB cDNA was inserted into the p*Kpn*I-CMV-sv40pA at *Cla*I and *Xba*I sites, the resultant plasmid was called p*Kpn*I-CMV-KRAB-sv40-pA. Then the TRE-PminCMV fragment derived from pTRE2pur was ligated into the pAd5-E1 shuttle at *Xho*I and *EcoR*I sites, and the obtained vector was called pAd5E1-TRE-PminCMV-shuttle. Using a similar strategy, the vector pAd5E1-TRE-PTminCMV-shuttle, which contains the Tight-PminCMV fragment derived from the pTight-PminCMV instead of the PminCMV element at the *Kpn*I and *EcoR*I sites, was obtained. Then the *Kpn*I site was removed from both plasmids. The plasmid pAd5-CMV-rtTA-TRE-pminCMV-shuttle was generated by inserting the fragment CMV-rtTA-sv40pA from pKpnI-CMV-rtTA-sv40pA, which was digested by *Kpn*I and *Xho*I into the *Kpn*I and *Sal*I sites of pAd5E1-TRE-pminCMV-shuttle. The plasmid pAd5-CMV-rtTA2S-M2-TRE-pminCMV-shuttle was obtained by the same method.

pCMV-PminCMV-rtTA2S-M2-sv40pA was generated by inserting the fragments PminCMV and rtTA2S-M2 into *Xho*I-*EcoR*I-*Spe*I sites of the pAd5-E1 sequentially. pAd5-Bi-Tet-On was generated by inserting the fragment PminCMV-rtTA2S-M2-sv40pA derived from the pCMV-PminCMV-rtTA2S-M2-sv40pA into *Not*I and *Xho*I sites of the pAd5E1-TRE-pTminCMV-shuttle in the opposite direction. The pAd5-KRAB-Bi-Tet-On was generated by cloning the fragment CMV-TetR-KRAB derived from pKpnI-CMV-KRAB-sv40pA into the *Kpn*I and *Not*I sites of pAd5-Bi-Tet-On. pAd5-CMV-rtTA-TRE-PminCMV contained two expression cassettes with the two promoters facing the same direction. In this system, rtTA was used as a transactivator and TRE-PminCMV controlled the target gene expression. In contrast to the pAd5-CMV-rtTA-TRE-PminCMV system, pAd5-CMV-rtTA2S-M2-TRE-PminCMV included rtTA2S-M2 rather than rtTA. pAd5-Bi-Tet-On was a bidirectional system, in which two promoters were placed on both sides of TRE in the opposite direction, and rtTA2S-M2 was expressed by the left cassette, which could be activated by the products of itself. pAd5-KRAB-Bi-Tet-On was similar to the pAd5-Bi-Tet-On system but a TetR-KRAB expression cassette was inserted at the 5′-terminal of the system.

### Luciferase activity assay

A dual-luciferase system was used to normalize cell number and transfection efficiency. HEK293 cells or CHO-K1 cells were plated into 24-well plates until the cell density reached 70%. Then the vectors and the Renilla luciferase as internal reference plasmid pRL-CMV (Promega, Madison, WI) were co-transfected into the cells using Lipofectamine™ 2000 Reagent (Invitrogen, Carlsbad, CA) according to the manufacturer’s protocol. Four hours later, the cells were cultured in fresh medium in the presence of 2 μg of Doxycycline/ml (Dox, Sigma-Aldrich, St. Louis, MO); 48 hours post-transfection, the cells were collected for a luciferase activity assay using a dual-luciferase assay kit (Promega, Madison, WI). The normalized luciferase activity was obtained by using the formula: Normalized luciferase value = Fly luciferase value/Renilla luciferase value.

### Adenovirus production

An adenovirus expressing sTRAIL in the E1 region and controlled by a Tet-On promoter was produced by co-transfecting *Pac*I linearized p-Ad5-KRAB-Bi-Tet-On-sTRAIL and the adenoviral backbone carrying an eGFP expression cassette in the E3 region into HEK293 cells grown in 60 mm dishes. Ten days post-transfection, the viral lysates were harvested and further propagated in HEK293 cells and were purified by cesium chloride gradient methods. The resultant adenovirus was named Ad5-KRAB-Bi-Tet-On-sTRAIL-eGFP. Ad5-KRAB-Bi-Tet-On-sTRAIL-eGFP carrying an eGFP expression cassette in the E3 region under the control of a CMV promoter was prepared as previously described [17]. The virus particle titers were detected by spectrophotometry at an absorbance of 260 nm.

### The assay of cell growth inhibition *in vitro*

SW480 cells (1 × 10^4^/well) were seeded into a 96-well plate the day prior to transduction with adenoviruses (250 multiplicity of infection (MOI)). Four hours post-transduction, the cells were cultured in fresh medium in the presence or absence of 2 μg Dox/ml. Cell growth inhibition was assayed 72 h post infection by an MTT (3-(4, 5-dimethylthiazol-2-yl)-2, 5-diphenyltetrazolium bromide) assay (Sigma, Saint Louis, MO), which was conducted according to the manufacturer’s protocol. MTT assay results were determined by measuring OD values at A570. U87 MG cells were transduced with different adenoviruses with 100 MOI.

### Statistics

Results are reported as the mean ± standard deviation (SD) and statistical analyses were performed using the Statistical Package for the Social Sciences (SPSS) software, version 13 (SPSS Inc., Chicago, Illinois). Differences between control and experimental groups were analyzed using one-way analysis of variance between groups (ANOVA/LSD), and a P-value of 0.05 or less indicated a statistically significant difference.

## Results

### Generation of a novel adenoviral E1 shuttle-based, all-in-one Tet-On system with an autoregulatory loop

**Figure 1:**
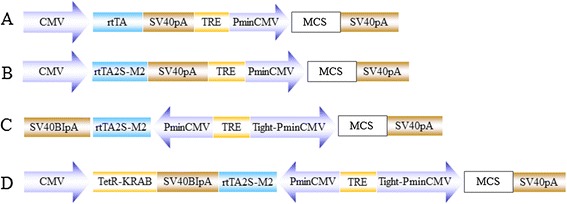
**Illustrations of different Tet-On inducible expression systems in the context of Ad5 E1 shuttle vector. (A)** pAd5-CMV-rtTA-TRE-PminCMV contains two expression cassettes with rtTA and the transgene controlled by two different promoters, pCMV and TRE-PminCMV, respectively, in the same direction. **(B)** pAd5-CMV-rtTA2S-M2-TRE-PminCMV is similar to pAd5-CMV-rtTA-TRE-PminCMV except that rtTA was replaced by rtTA2S-M2. **(C)** pAd5-Bi-Tet-On contains two promoters placed on both sides of TRE, forming an autoregulatory loop. **(D)** pAd5-KRAB-Bi-Tet-On contains an additional TetR-KRAB expression cassette compared to pAd5-Bi-Tet-On.

The major problem with an adenoviral vector carrying a Tet-On system is the suboptimal regulation of transgene expression. To address this problem, we first constructed three shuttle vectors, pAd5-CMV-rtTA-TRE-PminCMV (Figure 1A), pAd5-CMV-rtTA2S-M2-TRE-PminCMV (Figure 1B), and pAd5-Bi-Tet-On (Figure 1C). Based on these vectors, we then constructed an adenoviral E1 shuttle-based, all-in-one Tet-On system with a bidirectional autoregulatory loop, named pAd5-KRAB-Bi-Tet-On (Figure 1D). The final vector contained rtTA2S-M2, a new version of rtTA that has minimal basal activity and increased doxycycline sensitivity, and a tetracycline-controlled transcriptional silencer, TetR-KRAB.

### Improved regulational properties of the novel Tet-On system

**Figure 2:**
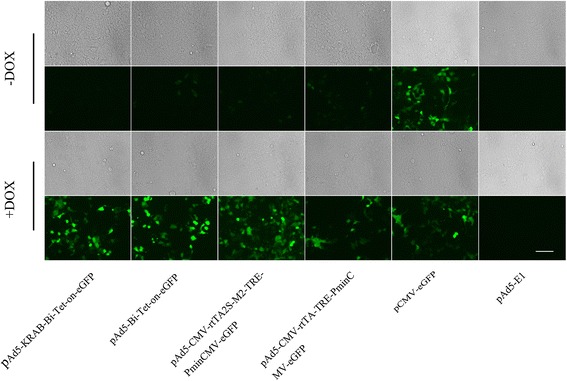
**Regulational expression of eGFP by the novel Tet-On system.** HEK293 cells were transfected with different inducible expression systems as indicated and were maintained in the presence or absence of 2 μg Dox/ml. pCMV-eGFP and pAd5-E1 were used as positive and negative controls, respectively. Forty-eight hours after transfection, images were taken with a fluorescent microscope (200 × view field).

The novel Ad5-KRAB-Bi-Tet-On system was first evaluated by expressing the reporter eGFP in HEK293 cells. Compared to controls, pAd5-KRAB-Bi-Tet-On-eGFP displayed minimal eGFP expression in the absence of Dox and a similar expression level of eGFP in the presence of 2 μg Dox/ml (Figure 2).

**Figure 3:**
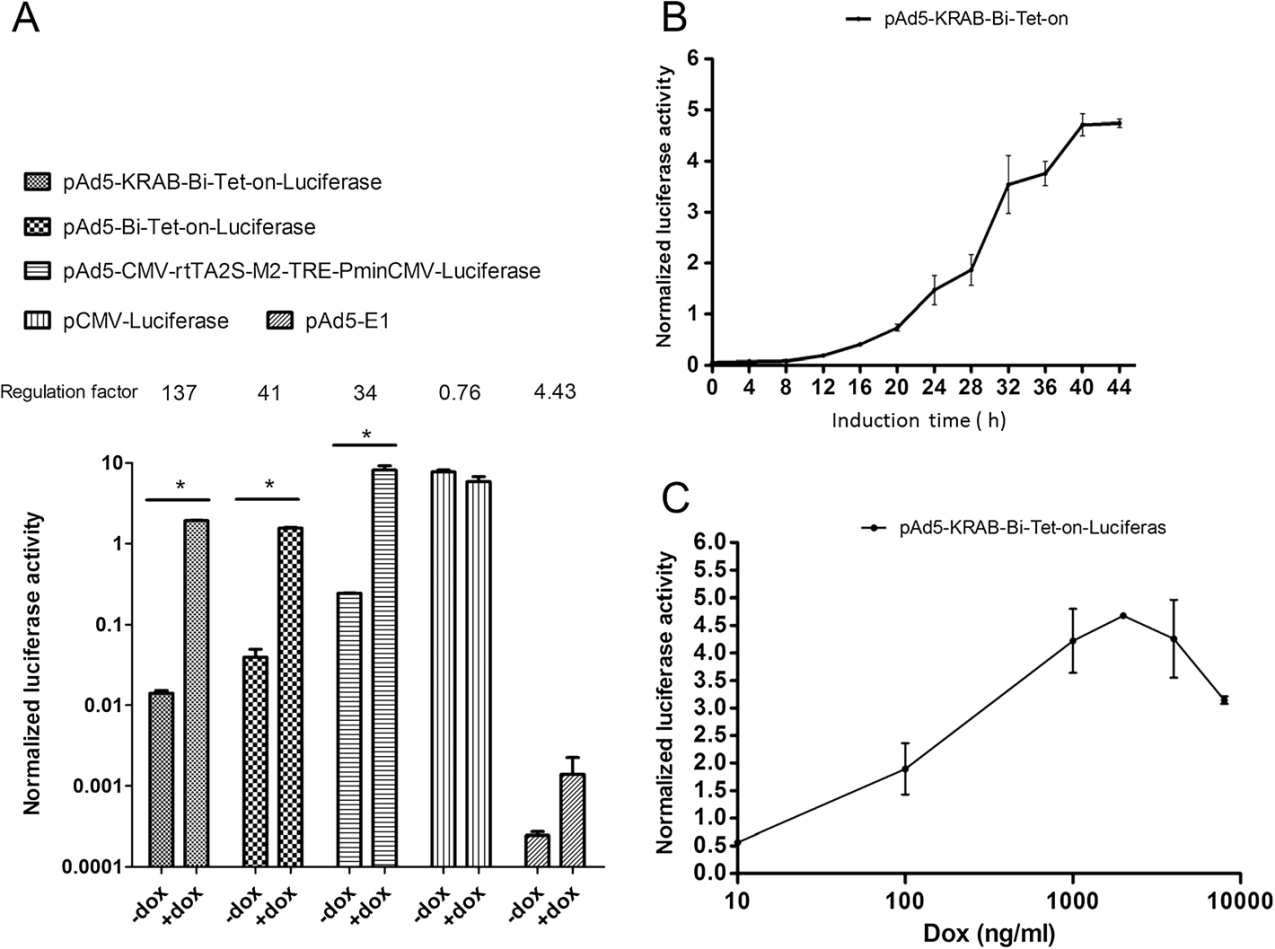
**Regulational luciferase expression by the novel Tet-On system. (A)** The comparison of different Tet-On systems. 293 cells were transfected with different Tet-On systems as well as the negative control pAd5-E1 and the positive control pCMV-Luciferase, in the presence or absence of Dox (2 μg Dox/ml). Forty-eight hours post-transfection, the activities of firefly luciferase and the internal control, Renilla luciferase, were quantified. The ratio of firefly luciferase to Renilla luciferase activity (FL/RL) was used to represent normalized luciferase activity. Experiments were repeated three times; *P < 0.05. **(B)** Time course of luciferase expression by pAd5-KRAB-Bi-Tet-On-Luciferase following Dox induction. CHO-K1 cells were transfected with pAd5-KRAB-Bi-Tet-On-Luciferase in the presence of 2 μg Dox/ml. Cells were collected every 4 h and normalized luciferase activities were obtained. Experiments were repeated three times. Data represent mean ± SD. **(C)** Dose–response curve demonstrating regulational expression of luciferase by the pAd5-KRAB-Bi-Tet-On vector. CHO-K1 cells were transfected with pAd5-KRAB-Bi-Tet-on-Luciferase in the presence of Dox at the final concentrations of 10 ng/ml, 100 ng/ml, 1000 ng/ml, 2000 ng/ml, 4000 ng/ml, 8000 ng/ml, and 10000 ng/ml. Forty-eight hours post-transfection, the cells were collected, and normalized luciferase activities were obtained. Experiments were repeated three times. Data represent mean ± SD.

The two most important criteria for evaluating a regulation system are the regulation factor (the ratio of maximum transgene expression level to minimum transgene expression level) and the maximum induced transgene expression level. To quantify these two criteria in the novel system, a reporter gene luciferase was cloned into each plasmid. The regulation factors of pAd5-CMV-rtTA2S-M2-TRE-PminCMV-luciferase, pAd5-Bi-Tet-On-Luciferase and pAd5-KRAB-Bi-Tet-On-Luciferase were 34, 41 and 137, respectively (Figure 3A). The normalized basal luciferase production of each of the three vectors in the absence of Dox was 0.2420 ± 0.0017, 0.0394 ± 0.0101 and 0.0141 ± 0.0011, and the induced (maximum) luciferase production in the presence of 2 μg Dox/ml was 8.1956 ± 1.0734, 1.5646 ± 0.0324 and 1.9226 ± 0.0332, respectively (Figure 3A). To our knowledge, pAd5-KRAB-Bi-Tet-On is the first reported adenoviral shuttle-based, all-in-one Tet-On system with a bidirectional autoregulatory loop that shows optimal regulated transgene production (the generally accepted standard is > 30). Additionally, as expected, TetR-KRAB in pAd5-KRAB-Bi-Tet-On further reduced the leakage of transgene expression in the absence of Dox (Figure 3A).

Next, we evaluated pAd5-KRAB-Bi-Tet-On’s responsiveness to Dox. pAd5-KRAB-Bi-Tet-On-Luciferase was transfected into CHO-K1 cells followed by the addition of Dox in different concentrations. Luciferase activity was measured in cell lysates every 4 h. The induction of luciferase expression from pAd5-KRAB-Bi-Tet-On-Luciferase was both time-dependent (Figure 3B) and dose-dependent (Figure 3C), with the maximal level of luciferase expression achieved with 2 μg Dox/ml after 40 h of induction.

### Adenoviral vector encoding sTRAIL under the control of the novel all-in-one Tet-On system

To investigate whether the novel Tet-On system could be suitable for regulating therapeutic genes in anti-tumor approaches, we constructed an adenovirus that carries soluble TRAIL under the control of our novel system. TRAIL is an anticancer agent that selectively induces apoptosis in a variety of cancer cells [18,19]. However, there is evidence that TRAIL has potential toxicity in some normal human cells [20-23]. Therefore, it is essential that the expression of TRAIL be strictly and consistently regulated in gene therapy protocols, so its concentration is within a ‘therapeutic window’ that maximizes treatment effects and minimizes the deleterious consequences of overexpression.

As a proof-of-principle, pAd5-KRAB-Bi-Tet-On, the adenoviral shuttle vector carrying the inducible expression system, was restricted by *Xho*I and *Xba*I followed by ligation with a sTRAIL fragment. The resultant plasmid was called pAd5-KRAB-Bi-Tet-On-sTRAIL. Then we co-transfected *Pac*I-linearized pAd5-KRAB-Bi-Tet-On-sTRAIL and *Pac*I-digested adenoviral backbone carrying an eGFP expression cassette in the E3 region into HEK 293 cells, to generate the recombinant adenovirus Ad5-E1-KRAB-Bi-Tet-On-sTRAIL-E3-eGFP. In addition, a non-inducible adenovirus carrying sTRAIL, named Ad5-E1-sTRAIL-E3-eGFP, was generated as a control adenovirus.

After propagation in the packaging cell line HEK293 (in the absence of Dox), the yield of Ad5-KRAB-Bi-Tet-On-sTRAIL was evaluated. Interestingly, it was found that the titer / infectious unit (IU) of Ad5-KRAB-Bi-Tet-On-sTRAIL was only about 35% of the control Ad5-E1-sTRAIL-E3-eGFP (Table 1). The lower viral particle/IU of the virus compared to that of the control might be attributable to the improved Ad packaging (due to the low basal level) and hence low cytotoxicity of sTRAIL.

**Table 1:** **The comparison of two types of viruses in titer and activity**

**Name of virus**	**Titer (pt/mL)**	**Activity (IU/mL)**	**Titer/Activity (pt/IU)**	**Titer/Activity (mean ± SD)**
Ad5-E1-KRAB-Bi-Tet-On-sTRAIL-E3-eGFP	1.9 × 10^12^	2.5 × 10^10^	76	55.67 ± 20.00
	3.0 × 10^12^	5.5 × 10^10^	55	
	1.8 × 10^12^	5.0 × 10^10^	36	
Ad5-E1-sTRAIL-E3-eGFP	2.2 × 10^12^	1.0 × 10^10^	220	160.33 ± 51.73*
	1.6 × 10^12^	1.25 × 10^10^	128	
	2.0 × 10^12^	1.5 × 10^10^	133	

*P < 0.05.

**Figure 4:**
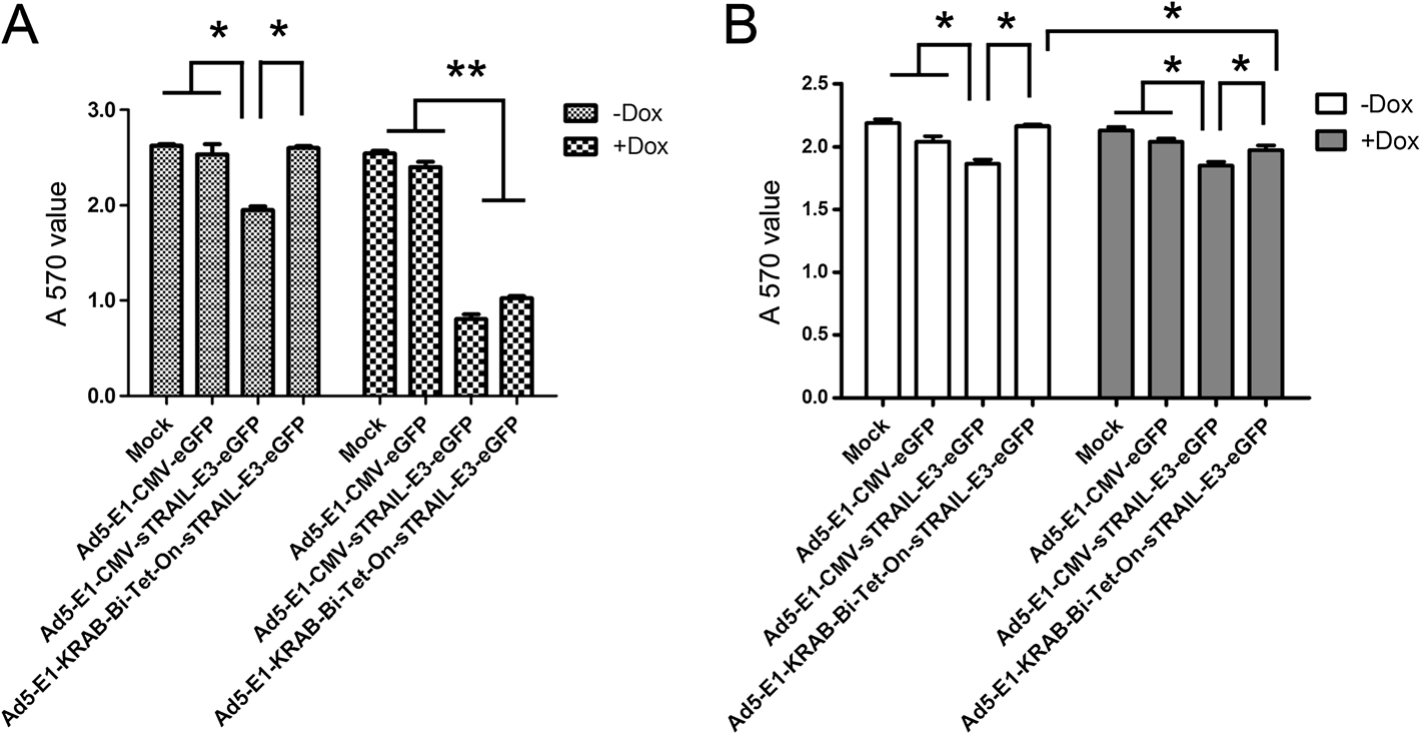
**Growth inhibitory effects of adenovirus encoding sTRAIL cells under the control of different Tet-On systems. (A)** SW480 cells were treated with PBS (mock), Ad5-E1-eGFP, Ad5-E1-sTRAIL-E3-eGFP and Ad5-E1-KRAB-Bi-Tet-On-sTRAIL-E3-eGFP, respectively, at 250 MOI in the presence or absence of Dox. **(B)** U87 MG cells were treated similarly but with a less dose of virus at 100 MOI. Cell growth inhibition was analyzed by MTT assay three days post-transduction. Experiments were repeated four times; *P < 0.05, **P < 0.01.

SW480 cell lines were then transduced with Ad5-E1-eGFP, Ad5-E1-sTRAIL-E3-eGFP and Ad5-E1-KRAB-Bi-Tet-On-sTRAIL-E3-eGFP (250 MOI). And U87 MG cells, which are TRAIL- insensitive [24-26], were used as the negative control. In order to achieve the similar infection efficiency to SW480 cells (approximately 90%), U87 MG cells was transduced with 100 MOI adenoviruses. Cell death was monitored by MTT assay three days post-transduction. No cytotoxicity was observed on SW480 cells with Ad5-E1-eGFP or Ad5-E1-KRAB-Bi-Tet-On-sTRAIL-E3-eGFP in the absence of Dox, whereas the cells transduced with Ad5-E1-sTRAIL-E3-eGFP or Ad5-E1-KRAB-Bi-Tet-On-sTRAIL-E3-eGFP in the presence of Dox showed increased cell death (Figure 4A). Similarly, Ad5-E1-eGFP or Ad5-E1-KRAB-Bi-Tet-On-sTRAIL-E3-eGFP had no effects on U87 MG cells in the absence of Dox; however, TRAIL-insensitive U87 MG cells showed less cell death compared with SW480 cells after being transduced with Ad5-E1-sTRAIL-E3-eGFP or Ad5-E1-KRAB-Bi-Tet-On-sTRAIL-E3-eGFP in the presence of Dox (Figure 4B). Interestingly, Ad5-E1-sTRAIL-E3-eGFP, the vector with stable sTRAIL expression, revealed greater suppressor activity on SW480 cells in the presence of 2 μg Dox/ml than in the absence of Dox (P < 0.01). It has been reported that some drugs could enhance TRAIL-induced apoptosis [24-28], although the underlying mechanisms remain unclear.

## Discussion

In this study, by utilizing a tetracycline-controlled transcriptional silencer (tTS or TetR-KRAB) and the latest generation rtTA, a single adenoviral vector carrying in its E1 region an all-in-one Tet-On system with an autoregulatory loop was successfully constructed to further improve the regulational properties of the Ad-mediated Tet-On system. This new system displayed improved Dox-inducible gene expression in terms of low basal expression, high induced expression and high responsiveness to Dox. To our knowledge, this is the first reported adenovirus-based, all-in-one Tet-On system with a large packaging size for transgenes up to 2.0 kb. Furthermore, the adenovirus that expresses soluble TRAIL under the control of this novel Tet-On system showed tumor-derived cells inhibitory activity in SW480 cells only under induced conditions.

A single Ad vector carrying the Tet-On regulation system could facilitate more practical and widespread application of the Ad vector. Some studies have used IRES-mediated bicistronic units for rtTA and tTS expression in the context of an Ad vector; however, no consistent regulational properties were observed in this system [15,16]. An Ad-mediated Tet-On system containing three expression cassettes (the gene of interest, rtTA2S-S2, and tTS) in the same vector was shown to display improved inducible gene expression [15]. However, the space for therapeutic transgenes was limited in this system. In addition, the bidirectional promoter for rtTA and transgene expression in this system did not show any Dox-induced luciferase production. In contrast, in our study, the adenovirus that carries a bidirectional promoter for rtTA2S-M2 and transgene expression displayed efficient inducible transgene expression with a regulational factor of up to 137, a low basal activity, and a maximum transgene expression level that is comparable to that of a CMV-driven non-inducible luciferase-expressing vector.

TRAIL can selectively induce apoptosis in many human cancer cells [18,19]. However, recent studies have shown TRAIL to be toxic to normal liver cells, keratinocytes, brain cells, prostate epithelial cells, as well as neutrophils [20-23]. Hence regulated TRAIL gene expression is critical in cancer gene therapy to prevent toxicity and optimize therapeutic outcomes. In addition, the regulated expression of sTRAIL in this study improved AD packaging probably due to the decreased cytotoxicity of sTRAIL. As expected, the sTRAIL-expressing adenovirus showed inhibition of Dox-induced tumor-derived cells growth.

Recently, genome editing techniques, including ZFN [29], TALEN [30] and CRISPR/Cas9 [31], have been widely used for gene functional analysis and gene therapy study. One of the key issues about these techniques is the spatiotemporal control of the nuclease expression to reduce off-target effects. The novel Tet-On system may be used to control the expression of ZFN, TALEN or Cas9 so that a desired level of nuclease expression can be reached at the right location and the right time, and therefore, the toxicity caused by nuclease can be minimized.

## Conclusions

We constructed a novel, single Ad vector carrying in its E1 region an all-in-one Tet-On system with an autoregulatory loop. This system displayed tight regulation of transgene expression *in vitro*, and thus has great potential for a variety of applications, including gene therapy and the study of gene function.
